# Role of personal aptitudes as determinants of incident morbidity, lifestyles, quality of life, use of health services, and mortality (DESVELA cohort): quantitative study protocol for a prospective cohort study in a hybrid analysis

**DOI:** 10.3389/fpubh.2023.1067249

**Published:** 2023-06-22

**Authors:** Ruth Martí-Lluch, Bonaventura Bolíbar, Joan Llobera, José A Maderuelo-Fernández, Rosa Magallón-Botaya, Álvaro Sánchez-Pérez, Ma José Fernández-Domínguez, Emma Motrico, Enric Vicens-Pons, Blanca Notario-Pacheco, Lia Alves-Cabratosa, Rafel Ramos, the DESVELA Cohort Investigators

**Affiliations:** ^1^Vascular Health Research Group of Girona, Institut Universitari per a la Recerca a l’Atenció Primària Jordi Gol i Gurina (IDIAPJGol), Girona, Spain; ^2^Parc Hospitalari Martí Julià, Institut d'Investigació Biomèdica de Girona (IDIBGI), Salt, Spain; ^3^Universitat Autònoma de Barcelona, Cerdanyola del Vallès, Spain; ^4^Network for Research on Chronicity, Primary Care, and Health Promotion (RICAPPS), Tenerife, Spain; ^5^Fundació Institut Universitari per a la recerca a l'Atenció Primària de Salut Jordi Gol i Gurina (IDIAPJGol), Barcelona, Spain; ^6^Balearic Islands Health Service (Ib-Salut), Primary Care Research Unit of Mallorca, Palma, Spain; ^7^Hospital Universitari Son Espases, GrAPP-caIB—Health Research Institute of the Balearic Islands (IdISBa), Palma, Spain; ^8^Unidad de Investigación en Atención Primaria de Salamanca (APISAL), Salamanca, Spain; ^9^Instituto de investigación Biomédica de Salamanca (IBSAL), Salamanca, Spain; ^10^Gerencia Regional de salud de Castilla y León (SACyL), Gerencia de Atención Primaria de Salamanca, Salamanca, Spain; ^11^Primary Health Care Research Group of Aragón (GAIAP), Institute for Health Research Aragón (IIS Aragón), Zaragoza, Spain; ^12^Department of Medicine, Psychiatry and Dermatology, University of Zaragoza, Zaragoza, Spain; ^13^Unidad de Investigación Atención Primaria de Bizkaia. Subdirección para la Coordinación de la Atención Primaria, Dirección General Osakiadetza, Vitoria, Spain; ^14^Grupo de Investigación en Ciencias de la Diseminación e Implementación en Servicios Sanitarios Instituto Investigación Biocruces, Baracaldo, Bizkaia, Spain; ^15^Ourense Health Area, SERGAS, Ourense, Spain; ^16^Centro de Saúde de Leiro, SERGAS, Leiro, Spain; ^17^I-Saúde Group, Hospital Álvaro Cunqueiro Bloque Técnico, South Galicia Health Research Institute, Vigo, Spain; ^18^Departamento de Psicología, Universidad Loyola Andalucía, Sevilla, Spain; ^19^Health Technology Assessment in Primary Care and Mental Health (PRISMA) Research Group, Parc Sanitari Sant Joan de Deu, Institut de Recerca Sant Joan de Deu, St Boi de Llobregat, Catalunya, Spain; ^20^Faculty of Nursing, Universidad de Castilla-La Mancha, Cuenca, Spain; ^21^Social and Health Research Center, Universidad de Castilla-La Mancha, Cuenca, Spain; ^22^Department of Medical Sciences, School of Medicine, Campus Salut, Universitat de Girona, Girona, Spain; ^23^Atenció Primària, Institut Català de la Salut, Girona, Catalonia, Spain

**Keywords:** primary health care, health promotion, chronicity, determinants of health, health behavior, morbidity, lifestyles, quality of life

## Abstract

**Introduction:**

The healthcare and well-being of the population depend on multiple factors and should adapt to societal changes. The opposite is also occurring; society has evolved concerning the individuals’ approach to their care, which includes participation in decision-making processes. In this scenario, health promotion and prevention become crucial to provide an integrated perspective in the organization and management of the health systems.

Health status and well-being depend on many aspects, determinants of health, which in turn may be modulated by individual behavior. Certain models and frameworks try to study the determinants of health and individual human behaviors, separately. However, the interrelation between these two aspects has not been examined in our population.

Our main objective is to analyze whether personal aptitudes related to behaviors are independently associated with the incidence of morbidity. A secondary objective will enquire whether these personal aptitudes are independently associated with lower all-cause mortality, enhanced adoption of healthy lifestyles, higher quality of life, and lower utilization of health services during follow-up.

**Methods:**

This protocol addresses the quantitative branch of a multicenter project (10 teams) for the creation of a cohort of at least 3,083 persons aged 35 to 74 years from 9 Autonomous Communities (AACC). The personal variables to evaluate are self-efficacy, activation, health literacy, resilience, locus of control, and personality traits. Socio-demographic covariates and social capital will be recorded. A physical examination, blood analysis, and cognitive evaluation will be carried out.

Several sets of six Cox models (one for each independent variable) will analyze the incidence of morbidity (objective 1); all-cause mortality and the rest of the dependent variables (objective 2). The models will be adjusted for the indicated covariates, and random effects will estimate Potential heterogeneity between AACC.

**Discussion:**

The analysis of the association of certain behavioral patterns and determinants of health is essential and will contribute to improving health promotion and prevention strategies. The description of the individual elements and interrelated aspects that modulate the onset and persistence of diseases will allow the evaluation of their role as prognostic factors and contribute to the development of patient-tailored preventive measures and healthcare.

**Clinical Trial Registration**: ClinicalTrials.gov, NCT04386135. Registered on April 30, 2020.

## 1. Introduction

In Spain, the population over 65 years of age is expected to increase by 10% in the next 50 years (1); and the total dependency ratio to grow from the current 54.2 to 72.2%. The aging of the population is associated with an increase in the number of people with chronic diseases (osteoarticular, cardiovascular, respiratory, mental, neurodegenerative, and cancer) and the resulting higher multimorbidity (two or more concurrent conditions) (2). This poses a great challenge to the health systems because the demand for health and social services escalates (3).

Chronic diseases are the leading cause of overall mortality and premature mortality in the world (they are related to 42% of the deaths occurring before 70 years of age). These conditions have an enormous impact on people’s daily life and their families, and represent a heavy burden on society (4). Among chronic diseases, the high prevalence of mental disorders is worth mentioning (5). Major depression, specifically, holds second place worldwide in terms of disability and disease burden (6). Mortality rates in people with mental health problems are up to double those in people without them. But importantly, most chronic diseases and their complications could be prevented through health promotion and primary prevention strategies; approximately 80% of cardiovascular diseases and 30% of all cancers could be averted (4).

Health promotion and prevention interventions play an essential role when considering the wellbeing of the population from a comprehensive perspective. According to the World Health Organization (WHO), health is a state of physical, mental, and social well-being that includes the ability to function, and not only the absence of disease or infirmity (7). From the perspective of health promotion, the Ottawa Charter defines health as a resource that allows people to lead individual, social, and economically productive lives (8). The general practitioner Jordi Gol stated that ‘health is an autonomous, supportive, and happy way of living’ (9). Furthermore, changes in society have also implied evolution in the role of the population concerning their care and decision making about their health.

In such a framework, health promotion is the process of equipping people with the necessary means to improve their health and exercise greater control over it (10, 11). According to the World Health Promotion Conference in Shanghai (2016), three areas within health promotion need priority: (1) good governance for health, (2) the promotion of healthy cities and communities, and (3) the reinforcement of health knowledge. These areas substantially coincide with the aims of the Strategy for Health Promotion and Prevention from the Spanish National Health System (SNS): to build healthy public policies, to create environments that support health and well-being, to support the empowerment of the individual through the development of personal skills, to reinforce community action, and to reorient the health services (12).

The health and well-being of both individuals and communities depends on the combination of many factors like where we live, our environment, genetics, income and education level, and family and social relationships. Notably, the access to and use of health care services have a limited impact; 80% of determinants of health are outside the influence of the health system (13).

These factors or determinants of health have been analyzed using various models. The Dahlgren-Whitehead model is one of the most widely used (14); it presents the main determinants of health as a range of concentric hierarchical layers where each layer determines the successive layers towards the center. Individuals are located in the center, with their non-modifiable characteristics such as age, sex, and genetic load. Around them, there are various layers of influences over health, such as lifestyles, social and community networks, living and working conditions, and socioeconomic, cultural and environmental conditions. Those factors that enhance the capacity of individuals to maintain health and well-being are defined as “Health Assets” according to the theory of salutogenesis (15). According to Marmot, control over one’s life and opportunities for full social participation are crucial aspects for health, well-being, and longevity (16).

Despite the endorsement of certain elements to improve the future well-being of the population (participation in the decision-making processes, the way people experience and cope with the diseases; and the ability to self-manage their own health and care), little is known about the role of personal determinants and individual aptitudes on the capacity to adopt health-promoting behaviors and respond appropriately to adverse situations. Several classic health-related behavior models and theories and the more recent integrative frameworks try to explain human behavior, the most widely used being the Theoretical Domains Framework (TDF) (17). This framework includes 12 domains derived from 33 theories and covers the main factors that influence behavior, namely knowledge, skills, social/professional role and identity, beliefs about capacities and consequences, memory, attention and decision processes, and social influences. Nevertheless, population-based studies on the predictive validity of the behavioral frameworks applied to health and well-being are lacking.

The importance of the development of personal aptitudes is stated in the Ottawa Charter (8) as one of the bases for establishing health public policies. The efforts to attain such a development should be directed towards providing information and health education and improving the abilities essential for life. This, in turn, would increase the options available for the population so they could exercise higher control over their health and the environment that influences it (8). The evaluation of personality traits includes a whole set of psychological and behavioral characteristics and the internal organization, which make different persons act differently when facing a similar circumstance (18). Certain personality traits are related to harmful behaviors, physical and mental health problems, lower longevity, and more mortality from all causes (19–23).

Some personal aptitudes stand out among those with higher potential impact on health improvement, quality of life, or reduced use of health services: personality traits, locus of control (LOC), self-efficacy, resilience, activation, and health literacy.

The locus of control (LOC) is defined as the extent to which individuals hold agency regarding the events that occur in their life. It can be internal—when the individuals believe that events in their lives are due to their own actions, attitudes, or behaviors; or external—when people believe it is the result of luck, chance, destiny, or the decisions of others (24). The presence of an internal LOC has been associated with a better perception of general health, a lower perceived burden of the diseases (25, 26), and a positive attitude towards health promotion and primary prevention activities (27). Regarding the workplace, the presence of an internal LOC is associated with higher job satisfaction and well-being, better job performance, and lower levels of stress (28). An external LOC has been associated with a worse health status (29), more use of the emergency services and hospital admissions (30), a higher risk of developing cardiovascular diseases (25) and other chronic diseases (31), and a worse physical and psychological health status (32).

Self-efficacy refers to the feeling of confidence in one’s abilities to adequately manage certain stressors in life (33). High self-efficacy was related to better mental function (34); better memory levels, speed of thought, and intelligence (35, 36); and a higher probability of acquiring healthy lifestyles (37). Low self-efficacy has been associated with anxious personality disorder (38).

Resilience is a dynamic process of positive adaptation to stress and adversity regarded as a protective factor against mental problems (39). A high degree of resilience has been considered a protective factor against mental diseases (40). Some authors have even suggested that it is a form of “mental immunity” (41). Resilience is also related to better cognitive function in older adults, specifically, greater verbal fluency and speed of perception (42).

Activation is defined as the capacity and ability to manage one’s personal condition, collaborate with the health provider to maintain one’s own health and wellbeing, access adequate and high-quality care, and prevent health deterioration (43). The Patient Activation Measure (PAM) allows evaluation of the self-knowledge, motivation, and aptitude to manage one’s own health (43). Activation is a tool that allows individuals to reach and maintain healthy lifestyles, and optimize their quality of life (44). Higher activation levels are associated with people with better self-healing capacity, better health status, and lower use of health services (45, 46).

Health literacy refers to the health knowledge of the population, their motivation and individual abilities to understand and make decisions related to the promotion and maintenance of their health (47). Adequate health literacy levels have been associated with healthy lifestyle behaviors, such as eating five servings of fruit and vegetables per day or being a non-smoker-regardless of age, educational level, sex, ethnicity, or income (48, 49). Low health literacy can hinder health self-care and be related to a higher incidence of chronic diseases (50). From a health and social perspective, the improvement of health literacy is an unavoidablechallenge.

To date, we have not identified longitudinal studies that delve into the analysis of all these personal aptitudes and their impact on health in our population. And yet, consideration of the interrelation of personal aptitudes and determinants of health is essential. Even more, there is solid evidence to support the association of socioeconomic, cultural, and environmental determinants with lifestyles, certain risk factors, and diseases (51). In the framework of the WHO 25 × 25 strategy, a recent meta-analysis of 48 cohorts that included 1.7 million people compared persons with low versus high socioeconomic status. Overall mortality rates were higher in the first group (hazard ratio, 95% confidence interval of 1.42, 1.38–1.45 in men, and 1.34, 1.28–1.39 in women), who also had those lifestyles that caused higher premature mortality (52). Other recent findings have shown that social and emotional support can protect health and well-being. However, further research should explain the reasons for this association, and understand its context and mechanisms (53). One of the most highlighted social determinants is the social capital, which refers to the resources available to individuals and groups through social networks (54, 55). Greater social capital has been associated with a better subjective perception of health and well-being (56, 57). Several observational studies indicated that higher social capital is a protective factor against mental and physical health, and mortality (58–60). Another conditioning factor is the working environment (13). Work stress has been associated with worse health status (61), increased risk of depressive disorders (62, 63), sleep disturbances (64), coronary heart disease (65), musculoskeletal pathology (66), alterations of lipid metabolism, and increased metabolic syndrome markers (67).

We believe that the analysis of the causal relationship between personal aptitudes and the adoption of healthy lifestyles, improvements in self-management of chronic conditions, quality of life, incidence and control of risk factors, incidence of chronic diseases and mortality is of special interest, and the creation of a cohort from Primary Health Care (PHC) is a most appropriate framework.

This project will contribute to provide essential knowledge for enabling and promoting the design of individualized interventions adapted to personal abilities and the evaluation of their role as prognostic factors. The interventions would also aim at improving the aptitudes that can be modified, such as health literacy, and the impact of these modifications may be assessed. Identification of the key determinants of multimorbidity is essential for the development of effective strategies for the healthcare and well-being of the person (2).

Accordingly, we propose to conduct an extensive investigation, with a holistic approach, on the determinants of health. Special focus will be given to the influence and effects of factors that determine individual behavior; a gender perspective and other aspects of social inequality will be included in the design and analysis. The description of these individual determinants involved in behaviors and their relation to social determinants, lifestyles, risk factors, chronic diseases and mortality are of paramount interest to our evolving societies and health systems.

### 1.1. Study objectives and hypotheses

This project was designed as a hybrid study, composed of a quantitative part (exposed in this article) and a qualitative part (published in another article).

The quantitative part of the DESVELA cohort has the following objectives:

1. To analyze whether personal aptitudes related to certain behaviors (self-efficacy, activation, health literacy, resilience, locus of control, and personality traits) are independently associated with the incidence of morbidity.
2. To analyze whether personal aptitudes related to certain behaviors (self-efficacy, activation, health literacy, resilience, locus of control, and personality traits) are independently associated with lower all-cause mortality, improved engagement in healthy lifestyles, higher quality of life, and lower utilization of the health services during follow-up.

We hypothesize that personal aptitudes that are related to positive behaviors (a higher self-efficacy, activation, health literacy, resilience, an internal locus of control, and positive personality traits) will be independently associated with a lower incidence of morbidity. We also hypothesize that personal determinants related to positive behaviors (a higher self-efficacy, activation, health literacy, and resilience, an internal locus of control, and positive personality traits) will be independently associated with all-cause mortality, engagement in healthy lifestyles, higher quality of life, and more optimal use of the health services during follow-up.

## 2. Materials and methods

### 2.1. Aim, design and study setting

**Figure 1:**
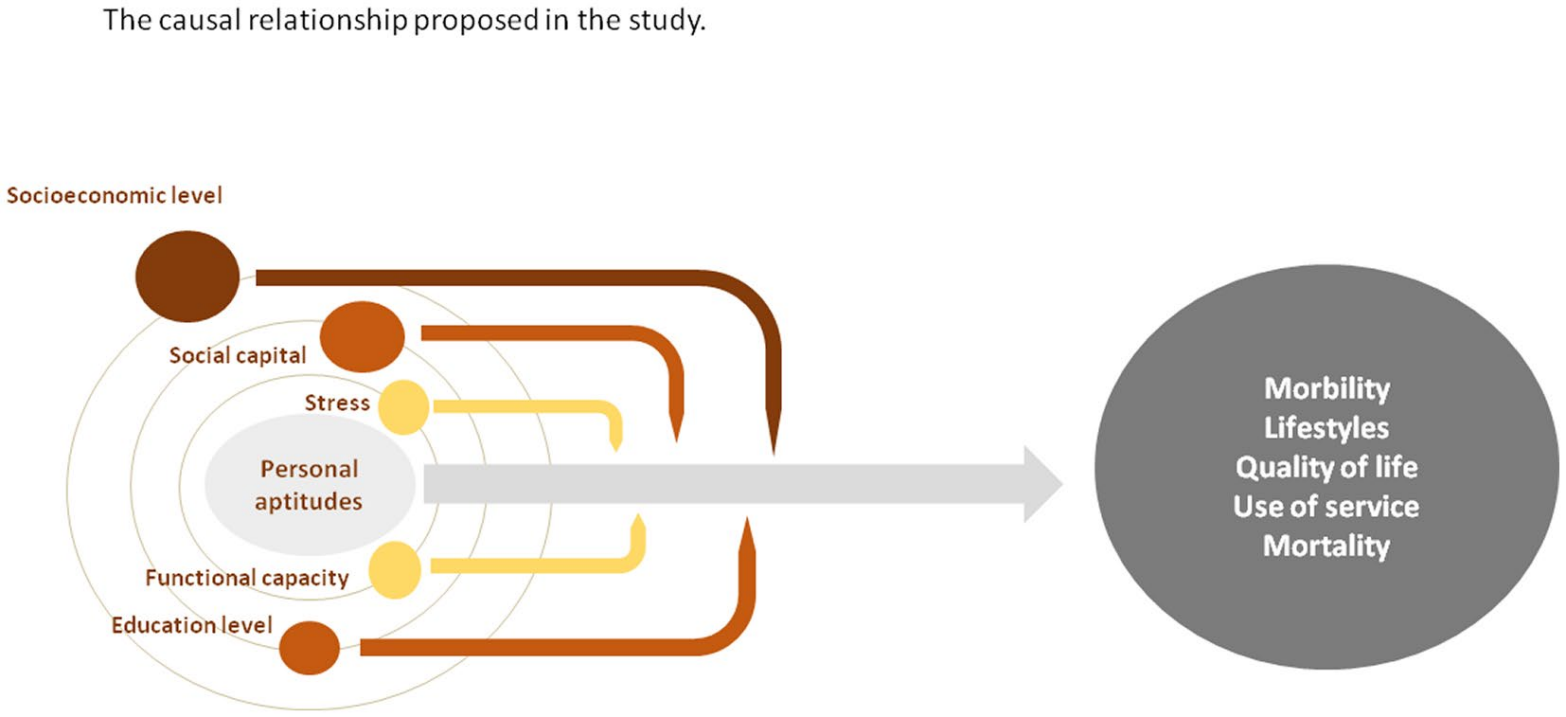
Diagram showing the causal relationship proposed in the study between the independent variables (personal aptitudes), the dependent variables (morbidity, lifestyles, quality of life, use of services and mortality) and the modulating effect of the covariates.

We aim to evaluate the influence of personal aptitudes on lifestyles and quality of life, the incidence of the most relevant health problems, the utilization of the health services, and all-cause mortality (Figure 1).

This is a multicenter study for the creation of a prospective cohort of persons assigned to Primary Healthcare (PHC) centers within nine autonomous communities (AACC; Catalonia, the Basque Country, Castilla y León, Aragón, Galicia, the Balearic Islands, Castilla La Mancha, Andalusia and Madrid). Follow-up examinations and evaluations will be at 5 and 10 years from the entry date.

### 2.2. Participants

We will include persons aged 35–74 years assigned to the above-mentioned PHC centers, selected by random sampling. Exclusion criteria will apply to persons with a terminal condition or institutionalized at the time of recruitment; persons with intellectual disabilities, dementia, or language difficulties; and persons who plan to move out of Spain within 5 years from study initiation.

### 2.3. Sample size

The sample size was computed using the GRANMO sample size calculator. We considered the estimation of the relative risk (RR) for a cohort study (using the Poisson approximation), with 10 years of follow-up and a rate of loss to follow-up of 30%, accepting an alpha risk of 0.05 and a beta risk of 0.2 in a bilateral contrast. The incidence of morbidity was considered as the main dependent variable and was around 60% in a previous analysis with 10.14 years follow-up (2). Health literacy was considered as the main independent variable. According to the questionnaire HLS-EU, 58,3% of the Spanish population has an inadequate or problematic level of health literacy (47). Low levels of health literacy have been linked to poorer physical and mental health outcomes, increased use of health services (68–70), and higher all-cause mortality (71, 72). Taking all these parameters into account, a sample of 3,083 persons is required, 1,793 in the exposed group (inadequate or problematic health literacy, HLS-EU-Q16 score between 0 and 12) and 1,290 in the non-exposed group (HLS-EU-Q16 score between 13 and 16). The sample size has been calculated to detect a minimum RR of 1.1 at 10 years, and will allow the detection of a minimum RR of 1.2 at 5 years, which is lower than some of the figures reported in the literature (73).

### 2.4. Variables

All the questionnaires used in the study are validated in Spanish except for the LOC, which will be assessed by a single question not yet validated. Table 1 shows the name of the questionnaire, the number of items, and the citation.

**Table 1:** Validated questionnaires used in the study.

Optional self-administration in our study	Questionnaires	Number of items	Reference of validated version in Spanish
	*Independent variables* *(Personal aptitudes)*		
Yes	Sherer’s general self-efficacy scale (GSES-12)	12	(74)
No	Patient Activation Measure (PAM)	13	(46)
Yes	10-item Connor-Davidson Scale	10	(75)
No	Health Literacy (HLS-EU-Q16)	16	(76)
No	10-Item Big Five Inventory (BFI-10)	10	(77)
	*Covariables*		
Yes	Functional social support (Duke-UNC-11)	11	(78)
Yes	WHO Disability Assessment Schedule (WHODAS 2.0)	12	(79)
Yes	List of Threatening Events (LTE)	12	(80)
Yes	Effort-Reward Imbalance questionnaire (DER/ERI)	16	(81)
	*Dependent variables*		
No	Adherence to the Mediterranean diet (Predimed and Predimed Plus)	23	(82, 83)
Yes	International Physical Activity Questionnaire (IPAQ)	4	(84)
Yes	Pittsburgh Sleep Quality Index (PSQI)	11	(85)
Yes	Quality of Life (EuroQol 5D–5 L)	5	(86)
No	Generalized anxiety disorder (GAD-7)	7	(87)
No	Depression Test Questionnaire (PHQ-9)	9	(88)
No	Depression Risk Calculator (PredictD)	2 + 12	(89)

The independent variables in this study refer to personal aptitudes, will be measured at baseline, and are listed as follows with the corresponding measurement tools: self-efficacy, which will be measured with the Sherer’s general self-efficacy scale (74); activation, measured with the Patient Activation Questionnaire (PAM) (46); resilience, measured with the abbreviated version of the 10-item Connor-Davidson scale (75); health literacy, evaluated with the HLS-EU-Q16 literacy questionnaire (76); LOC, assessed with the statement “I feel that events in my life are often determined by factors that are beyond my control” which has 6 response options in a Likert-type scale; personality characteristics will be examined using the 10-Item Big Five Inventory (BFI-10) for determination of personality traits (77).

The dependent variables in this study are morbidity, all-cause mortality, adoption of healthy lifestyles, quality of life, and utilization of health services. The information regarding the main dependent variables will be recorded at baseline and during the follow-ups at 5 and 10 years through surveys, physical examination and review of the medical records. The presence of morbidities will be assessed through the diagnoses in the medical records and physical examination. The medical records will also be the source to assess all-cause mortality.

Regarding the physical examination, blood pressure of participants will be measured and hypertension defined following the recommendations and criteria of the Spanish Hypertension Society. The ankle-brachial index (ABI) will be measured with a Vasera device (Fukuda Denshi), and peripheral arterial disease considered when ABI < 0.9. Weight and height will be measured to obtain the body mass index (BMI), and overweight and obesity will be considered if 25 ≤ BMI < 30 and BMI > 30, respectively. Waist circumference will also be measured. Blood testing will be performed to measure fasting glucose levels, glycated hemoglobin (HbA1c), total, low and high-density lipoprotein cholesterol, triglycerides, and creatinine.

For each participant, morbidity will be recorded at baseline and during follow-up by considering all the active conditions in the medical records at the time of the visit. We will define incident morbidity as the onset of any of the following 17 group conditions, provided they are not present at baseline: hypertension, ischemic heart disease (angina or acute myocardial infarction), heart failure, cardiac arrhythmias, diabetes mellitus, ischemic stroke, peripheral arterial disease, chronic obstructive pulmonary disease, asthma, any type of arthritis, osteoporosis, any type of cancer, Parkinson’s disease, one or more affective disorders (depression, anxiety), one or more psychotic disorders (schizophrenia, psychosis, bipolar disorder), dementia (including Alzheimer’s disease), and obesity. If we detect any frequent condition at follow-up that has not already been included in these 17 proposed groups, we will add a new group category.

The evaluation of the participants’ mental health will also include the following: a questionnaire on the generalized anxiety disorder (GAD-7) (87), which evaluates the presence of symptoms; examination of the diagnostic criteria for major depression, using the PHQ-9 (88); and estimation of the risk of depression (89), in participants with no diagnosis of a major depressive episode.

We will evaluate the several lifestyles: adherence to the Mediterranean diet, assessed with the PREDIMED (82) and PREDIMED plus (83) questionnaires; type of diet, determined by asking the participants if they follow any specific diet (e.g., vegetarian, vegan); level of physical activity, assessed with the International Physical Activity Questionnaire (84); smoking habit, measured with the 4-question scale adapted from the WHO MONICA study (90); alcohol consumption, considering the total units during the past week and a question on the monthly frequency of excessive consumption (binge drinking) over the past year; and sleep, evaluated using the Pittsburgh Sleep Quality Index (85), the overall number of sleeping hours, usual bedtime, and waking-up time.

The quality of life will be determined using the EUROQol 5D-5L health questionnaire (86), and a question on self-perceived well-being. Finally, the utilization of health services for the last 12 months previous to inclusion into the study will be extracted from electronic health records. Where the research team may have no access to electronic health records, the utilization of health services will be assessed with a survey answered by the participants. The items will enquire on the number of visits (to the emergency department, primary care, and other specialties), hospital admissions, diagnostic tests, and pharmacological treatment (medication, dose, total daily dose, and duration).

Additionally, we will consider the following groups of covariates: sociodemographic, social, functional capacity, and stress level.

We will register the following sociodemographic variables: date of birth, sex, sex orientation, and gender identity (SOGI questions), marital status, nationality, and employment status. We will also consider occupational social class, defined with the educational level and occupation. Participants will answer questions on employment conditions (six items), domestic and care work (six items), income level and economic situation (four items), and housing and material situation.

The two social variables included in this study are the social capital and the functional social support questionnaire. To assess the social capital, we will use the harmonized questionnaire proposed by Blaxter et al. (91) where five dimensions are identified: perspectives about the local area, civic engagement, social and support networks, social participation, and reciprocity and trust (91, 92). To assess the functional social support we will use the validated Spanish version of DUKE-UNC-11 (78). The questionnaire evaluates two sub-scales: confidential support (7 items) and affective support (4 items).

The functional capacity of participants will be evaluated using the WHO Disability Assessment Schedule (WHODAS 2.0) (79), a 12-item self-administered questionnaire.

The stress level will be assessed by taking into account stressful life events, using the List of Threatening Experiences questionnaire (80); and also occupational stress, determined in employed persons at the time of recruitment using the Effort-Reward Imbalance questionnaire (81). There will also be five questions regarding the impact of COVID-19.

### 2.5. Data collection process

Once the project has been approved by the pertinent ethics committees, informative meetings will be held at the PHC centers to explain the project to health professionals so they can address any questions that may arise. Subsequently, assigned personnel (may vary according to the AACC) will be asked to prepare a list with the people ascribed to the PHC center who meet the inclusion criteria of the study and make a random selection of the necessary sample, oversampling by 30%, in order to be able to substitute participants that should be excluded. In some PHC centers, professionals will directly call possible participants to invite them to be included in the study. In other centers, permission will be requested from the professionals so that a person linked to the study makes the calls on their behalf in a centralized manner.

Participants recruitment: a letter will be sent by mail to the candidates with the study information sheet and 10 self-administered questionnaires: (1) the self-efficacy scale; (2) the resilience scale; (3) level of physical activity; (4) quality of life; (5) the sleep quality index; (6) a disability assessment; (7) occupational stress; (8) stressful life events; (9) the functional social support questionnaire; and (10) the SOGI questions. This letter will be sent to participants before or after the phone contact, as decided by each AACC. The person in charge of calling the participants will invite them to participate in the study and schedule a day and time to attend their PHC centers. If they accept, they will be asked to fill out the self-administered questionnaires and deliver them to a nurse at the PHC.

During the face-to-face visit, the person will be invited to sign the informed consent and clarify doubts, if any, about the information sheet and the study in general. In a confidential sheet, separate from the rest of the variables, the name, surnames, postal address, and contact telephone numbers will be recorded. A case identification number will be given. Study data will be collected and managed using REDCap (93, 94) electronic data capture tools hosted at *Fundació Institut Universitari per a la recerca a l’Atenció Primària de Salut Jordi Gol i Gurina* (IDIAPJGol).

**Figure 2:**
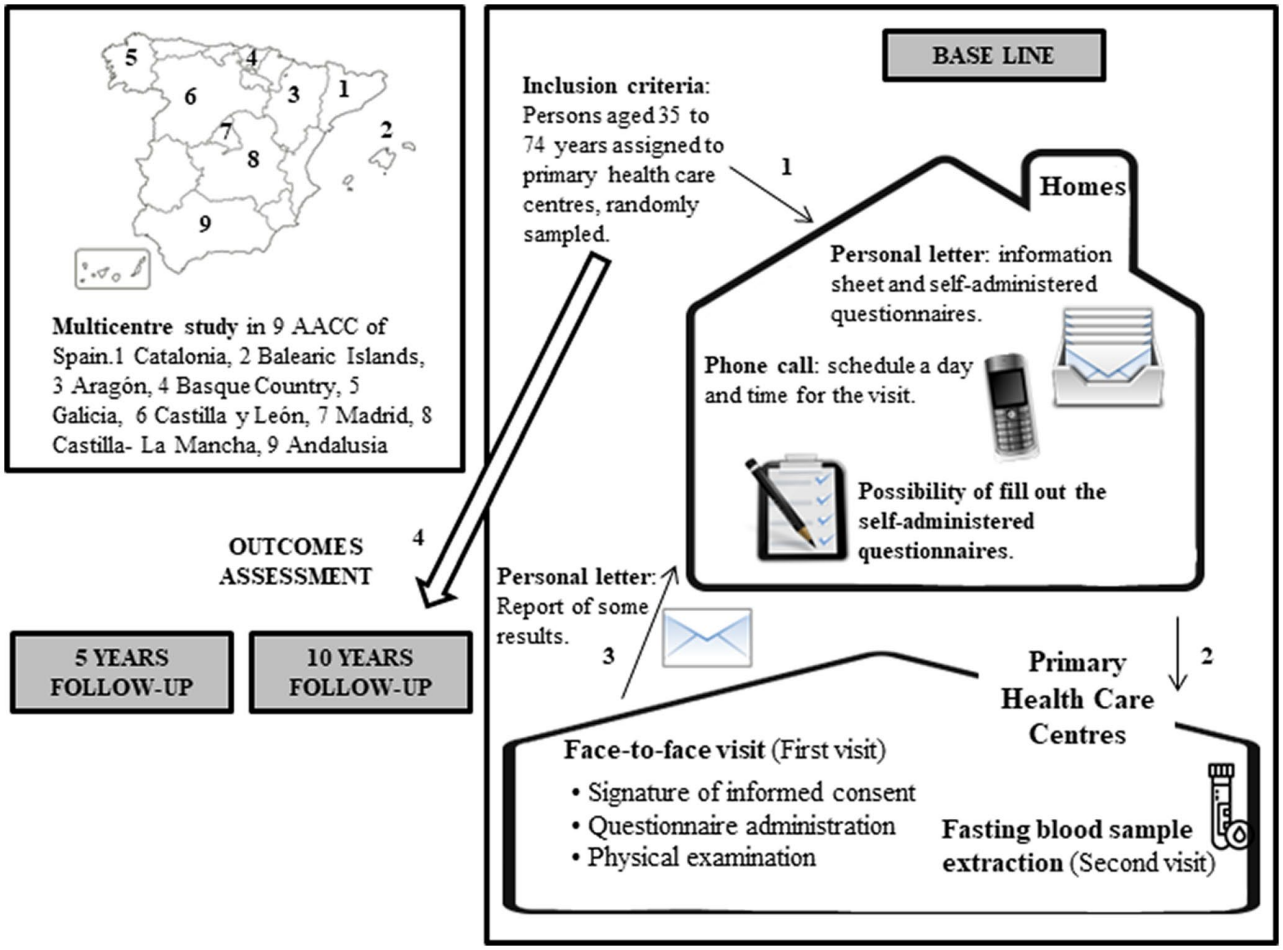
Data collection process with the actions that will affect the participants included in the study.

During the visit, participants will be invited to answer the questionnaires and the physical examination will be performed. The information collected in the self-administered questionnaires will be reviewed; if someone has not been able to fill them out, they will do it during the visit. On this first visit, a fasting blood sample extraction will be scheduled to determine (on a second visit) the parameters explained before. Finally, a letter with the examination results will be sent to each participant to allow consultation with the professionals from their health center if any abnormalities were detected. Figure 2 shows the data collection process with the actions that will affect the participants included in the study.

### 2.6. Statistical analysis

The statistical analysis will include a descriptive analysis. Percentages of the categorical variables will be calculated for each AACC. Continuous variables with a normal distribution will be described with the mean (standard deviation, SD); otherwise, the median (first and third quartile) will be used. The analyses will be stratified by sex. Bivariate analyses will be performed using the *t*-test for independent samples, or the Mann–Whitney test when required, to analyze continuous variables; and the Chi-squared test for proportions.

Objective one will analyze the incidence of morbidity, defined as the onset of any new condition out of the 17 considered. To this end, a Cox model will be built for each independent variable, and adjusted for the above-mentioned covariates. Potential heterogeneity between AACC will be estimated by introducing random effects in the model. Sensitivity analyses will examine loss to follow-up rates. Objective two will analyze the incidence of the rest of the dependent variables (except incidence of morbidity) using also Cox models, one for each of the six independent variables, adjusted for the covariates. We will calculate the incidence of all-cause mortality and its Kaplan–Meier, stratified by the categorical variables and AACC. Potential heterogeneity will be estimated, and sensitivity analyses performed as in objective one.

Before obtaining the follow-up information at 5 and 10 years, we will describe and analyze the gathered data at baseline. To this end, general linear multilevel models will be used to examine the association of the dependent variables (lifestyles, quality of life, and use of services), with the six independent variables, adjusted for the registered covariates (age, sex, occupational social class, social capital, functional capacity, and stress level). Potential disparities between AACC will be captured by including random effects in the models.

Statistical significance will be considered at *p*-values < 0.05. The analyses will be carried out using the Statistical Package for the Social Sciences (SPSS) version 26.0, and the R-software.

## 3. Discussion

Highly prevalent serious conditions should be one of the targets of research efforts. This is the case of chronic diseases, the leading cause of death in our population, and a major cause of disease burden, number of life-years lost, and resource consumption in our society. Chronic diseases and their consequences will be aggravated by an ongoing demographic trend that is predicted to linger on: the progressive ageing of the population (95, 96).

Health promotion and the strategies for disease prevention are particularly effective in the context of chronic diseases, because many of their associated risk factors could certainly be modified and prevented. This project aims to contribute to a framework on which efficient and safe interventions on health promotion and prevention could be developed, and the number of persons suffering these diseases in the future could be reduced. We need to promote and encourage the autonomy of people to carry out their activities, a constant care and improvement of their physical and psychological abilities, a delayed onset of early dependence, and the maintenance of their social environment. This would lead to a reduction in the burden of disease on society, a huge improvement in people’s quality of life, and also a reduction of the enormous cost on overloaded health systems.

The evidence generated in this study will add new knowledge to previous attempts at establishing comprehensive theoretical frameworks that explain people’s health behaviors, such as TDF (17) or the capability, opportunity, and motivation (COM-B) model (97, 98). The Primary Care context is the ideal environment to start this research due to the almost total similarity with the characteristics of the reference population. Moreover, it is where many of the indicators that we intend to analyze are developed and undertaken. Additional to the identification of the subjects, collection of all the baseline information and initial cross-sectional analysis, the longitudinal follow-up will explore the influence of these factors as predictors of lifestyles and the impact on multimorbidity, as reported in several systematic reviews (99, 100).

The conduct of this project will lay the basis for pioneering new methods in clinical practice, particularly for tailoring interventions according to the individual characteristics of each person, aiming to improve their acceptance and efficiency. Indeed, the project is directed towards extending personalized medicine, caring for and treating the person as a whole, and not only the disease. At the same time, it offers an opportunity to optimize the resources and sets a sustainable strategy that can be transferred into clinical practice through recommendations.

### 3.1. Study limitations

One of the chief difficulties in this study could be a low participation rate. A strategy to achieve adequate participation rates is to invite via phone call on behalf of the physician or nurse from their health center. Another approach would be to send an informative mail previous to the phone call so that when researchers contact potential participants, they already know the objective of the call and are more willing to listen to the invitation. In this project, we will follow both strategies. We have previous experience in carrying out cohort studies, with successful participation rates. Some of the measurements and administration of the questionnaires of this study require previous training, to ensure precise, comparable, and high-quality results. This challenge will be minimized by training the professionals that will perform them and controlling the quality of the periodically collected data. The measure of LOC in this study will be a single non-validated question. Finally, the survey will ask a considerable amount of questions, and thus the visits are at risk of being too long and tedious. To avoid this, some of the self-administered questionnaires will be attached to the above-mentioned informative mail. At home, participants will have plenty of time to answer them, before the visit. In previous studies, 60% of participants came to the visit with the questionnaires already answered, and this percentage could be higher if a reminder is given at the recruitment call.

## 4. Conclusion

Health promotion is a priority within the public health policy of developed and developing countries. Current knowledge describes the association of genetic, socioeconomic, cultural, and environmental determinants with lifestyles, risk factors, and diseases. However, little is known about the real effect of personal determinants on individual behaviors; these aptitudes may be connected with our personal capacity to adopt healthy lifestyles and respond suitably in the face of adverse situations. This project will fulfill the need for cohort studies on behavioral changes, maintenance of healthy behaviors, reduction of harmful habits, improvement in the self-management of chronic conditions, and increase in the quality of life related to health determinants. The collaborators in charge of this study present a holistic approach to examine health determinants with a special focus on individual behaviors. Description of these individual factors, their relation with social factors, lifestyles, and chronic diseases will be highly interesting and useful to the society and the national health system. This knowledge will allow the evaluation of their role as prognostic factors and the interaction with the rest of determinants to tailor interventions, and the design of interventions directly aimed at improving these individual capacities.

## Data availability statement

The original contributions presented in the study are included in the article/supplementary material, further inquiries can be directed to the corresponding author.

## Ethics statement

The studies involving human participants were reviewed and approved by Fundació Institut Universitari per a la recerca a l’Atenció Primària de Salut Jordi Gol i Gurina (reference number 19/150-P); Comité de Ética de la Investigación con medicamentos del Área de Salud de Salamanca (reference number PI 2020 02424); Andalusian Ministry of Health, Spain (reference number: 1260-M1-21); Comité de Ética de la Investigación de medicamentos de Euskadi (CEIm-E; reference number: PI2020185); Hospital Virgen de la Luz Clinical Reasearch Ethics Comiitte, Cuenca, Spain (Reference number 2019/PI2119). Research Central Commission of the Primary Care Assistance Management, Madrid, Spain. (Reference Number 07/21); Comité de Ética de la Investigación de la Comunidad Autónoma de Aragón (reference number: PI20/302); Galician Ministry of health, Spain: high impact study authorization (Reference number: 2021/047). The patients/participants provided their written informed consent to participate in this study.

## DESVELA Cohort Investigators

***Andalucía***. Universidad Loyola Andalucía: Emma Motrico, Irene Gómez-Gómez. Instituto de Investigación Biomédica de Málaga: Patricia Moreno-Peral, Sonia Conejo-Cerón, Juan Ángel Bellón. ***Aragón***. Fundación Instituto de Investigación Sanitaria Aragón: Rosa Magallon-Botaya, Fátima Méndez-López, Alejandra Aguilar-Latorre, Maria Beltrán-Ruiz, Bárbara Oliván-Blázquez, Marta Dominguez-García, Maria Isabel Rabanaque Hernandez, Eva María Andrés Esteban. ***Castilla-La Mancha***. Centro de estudios sociosanitarios: Blanca Notario Pacheco, Montserrat Solera Martínez, Lidia Lucas-de la Cruz, Miriam Garrido Miguel, María Martínez Andrés, María Eugenia Visier Alfonso, Irene Marcilla Toribio. ***Castilla y León***. Unidad de Investigación de Atención Primaria de Salamanca. José A Maderuelo-Fernández, Leticia Sierra-Martínez, Olaya Tamayo-Morales, Miriam Daniela García-Cubillas, Ana B Castro-Rivero, María D Martín-Santos, Carmen Castaño-Sánchez, Luis García-Ortiz. ***Catalunya***. Fundació Institut Universitari per a la recerca a l’Atenció Primària de Salut Jordi Gol i Gurina: Bonaventura Bolíbar, Ruth Martí-Lluch, Rafel Ramos, Marc Casajuana-Closas, Anna Berenguera, Constanza Jacques-Aviñó, Yudy Young-Silva, Lia Alves-Cabratosa, Lluís Zacarías-Pons, Anna Ponjoan, Eva Espigulé-Ribas, Francesc Ribas-Aulinas, Jordi Blanch, Èric Tornabell-Noguera, Anna Moleras-Serra. Parc Sanitari Sant Joan de Déu: Enric Vicens-Pons, Montserrat Gil-Girbau, Mari Carmen Olmos Palenzuela, María del Carmen Gallardo González, Mª Teresa Peñarrubia-María, Paula Arroyo-Uriarte. ***Comunidad de Madrid***. Centro de Salud Infanta Mercedes, Servicio Madrileño de Salud. Francisco Camarelles Guillem. ***Euskadi***. Instituto para la Investigación Sanitaria Biocruces Bizkaia. Jose María Aiarzaguena, Álvaro Sánchez Pérez, Sandra Garcia-Martinez, Usue Elizondo Alzola, Mónica Miranda de la Maza, Ainhoa Abrisketa Ullibarri, Mikel Rueda-Etxebarria. ***Galicia***. Instituto de de investigación Galicia Sur: Mª José Fernández Domínguez, Sabela Couso Viana, Roberto Fernández Alvarez, Ana Claveria Fontan, Ana Isabel Castaño Carou, Clara González Formoso, María Victoria Martín Miguel, Clara Guede Fernández, Macarena Chacón Docampo. ***Illes Balears***. Gerencia de atención primaria de Mallorca, Instituto de investigación sanitaria de las Islas Baleares: Joan Llobera Cànaves, Caterina Vicens, Maria J. Serrano-Ripoll, Laura Gallardo-Alfaro, Oana Bulilete, Christian Jean-Mairet Soler, David Medina-Bombardó, T Coll Benejam.

## Author contributions

RR and RM-L conceived the quantitative part of the study. RM-L and LA-C led the drafting of this manuscript. RM-L, JL, JM-F, RM-B, ÁS-P, MD, EM, EV-P, and BN-P obtained ethical approval from each institution. RM-L, RR, BB, JL, JM-F, RM-B, ÁS-P, MD, EM, EV-P, and BN-P advised and contributed to the study design. RM-L contributed to design the training program on the questionnaires and measurements. RR, RM-L, and JM-F developed the statistical analysis plan. RM-L, RR, and EV-P designed the economic components of the study and developed the economic analysis. All authors have revised the draft substantively, given approval of the submitted version (and any substantially modified version involving their contribution to the study), and agreed to be accountable for their own contributions and to ensure that all aspects of the study, including those in which they were not personally involved, are appropriately addressed.

## Funding

This study has been funded by Instituto de Salud Carlos III (ISCIII) with competitive grants for the period 2019–2022 through the Fondo de Investigación para la Salud (FIS), which is co-funded by European Regional Development Fund/European Social Fund “A way to make Europe”/“Investing in your future.” Project Grants codes are: P19/01285; P19/00997; P19/01140; P19/00147; P19/01076; P19/00434; P19/01459; P19/01314; P19/01264 and P19/00115. The coordinator group received a pre doctoral training contract in health research (PFIS-FI20/00270) from the 2020 caLL of the Strategic Action in Health 2017–2020. Investigation groups were also funded through the Research Network in Preventive Activities and Health Promotion in Primary Care (redIAPP), RD16/0007/0001; RD16/0007/0002; RD16/0007/0003; RD16/0007/0004; RD16/0007/0005; RD16/0007/0006; RD16/0007/0008; RD16/0007/0009; RD16/0007/0010 and RD16/0007/0012; and through the research grants on the call for the creation of Health Outcomes-Oriented Cooperative Research Networks (RICORS) co-funded with European Union-NextGenerationEU funds, allowing the creation of the Network for Research on Chronicity, Primary Care, and Health Promotion (RICAPPS) with the following references: RD21/0016/0001; RD21/0016/0003; RD21/0016/0005; RD21/0016/0009; RD21/0016/0010; RD21/0016/0012; RD21/0016/0018; RD21/0016/0022; RD21/0016/0025 and RD21/0016/0029. Additional grants: Gerencia Regional de Salud de Castilla y León (GRS 2306/B/21 and GRS 2356/B/21); Andalusian Ministry of Education and Science (PY20 RE 025). The funders had no role in the study design, writing of the report, or in the decision to submit the protocol for publication. All authors confirm that they worked independently from funders.

## Conflict of Interest

The authors declare that the research was conducted in the absence of any commercial or financial relationships that could be construed as a potential conflict of interest.

## Publisher’s note

All claims expressed in this article are solely those of the authors and do not necessarily represent those of their affiliated organizations, or those of the publisher, the editors and the reviewers. Any product that may be evaluated in this article, or claim that may be made by its manufacturer, is not guaranteed or endorsed by the publisher.

## Abbreviations

AACC: Autonomous Communities
ABI: Ankle-brachial index
BFI-10: 10-Item Big Five Inventory
BMI: Body Mass Index
COM-B: Capability, Opportunity and Motivation (COM-B) Model
IDIAPJGOL: Fundació Institut Universitari per a la recerca a l’Atenció Primària de Salut Jordi Gol i Gurina
IPAQ: International Physical Activity Questionnaire
LOC: Locus of Control
PAM: Patient Activation Measure
PHC: Primary Health Care
SNS: Spanish National Health System
SOGI: Sex orientation and gender identity
TDF: Theoretical Domains Framework
WHO: World Health Organisation
